# Blockade of potassium ion transports enhances hypotonicity-induced cytocidal effects in gastric cancer

**DOI:** 10.18632/oncotarget.20736

**Published:** 2017-09-08

**Authors:** Toshiyuki Kosuga, Atsushi Shiozaki, Michihiro Kudou, Yuzo Yamazato, Daisuke Ichikawa, Shuhei Komatsu, Hirotaka Konishi, Kazuma Okamoto, Katsutoshi Shoda, Tomohiro Arita, Ryo Morimura, Yasutoshi Murayama, Yoshiaki Kuriu, Hisashi Ikoma, Masayoshi Nakanishi, Hitoshi Fujiwara, Yoshinori Marunaka, Eigo Otsuji

**Affiliations:** ^1^ Division of Digestive Surgery, Department of Surgery, Kyoto Prefectural University of Medicine, Kyoto, Japan; ^2^ Department of Molecular Cell Physiology and Bio-Ionomics, Graduate School of Medical Science, Kyoto Prefectural University of Medicine, Kyoto, Japan; ^3^ Japan Institute for Food Education and Health, St. Agnes’ University, Kyoto, Japan

**Keywords:** gastric cancer, peritoneal lavage, peritoneal dissemination, potassium channel, regulatory volume decrease

## Abstract

**Background:**

Peritoneal lavage with distilled water has been used for surgeries of various cancers to reduce peritoneal recurrence. This study examined whether blockade of potassium ion transports enhances hypotonicity-induced cytocidal effects in gastric cancer (GC).

**Results:**

A potassium channel blocker inhibited the occurrence of regulatory volume decrease (RVD) induced by mild hypotonic stimulation, and significantly enhanced cytocidal effects on GC cells. Incubating MKN45 cells with hypotonic solutions containing a potassium channel blocker significantly reduced the formation of peritoneal metastases in nude mice.

**Methods:**

The three human GC cell lines (HGC-27, Kato III, and MKN45) were exposed to mild hypotonic solutions, and the effects of blockade of potassium ion transports during hypotonic stimulation on cell volume changes and cell viabilities were examined. In the *in vivo* study, MKN45 cells stimulated with mild hypotonic solutions were intraperitoneally injected into nude mice, and the effects of blockade of potassium ion transports during hypotonic stimulation on the formation of peritoneal metastases were evaluated.

**Conclusions:**

Blockade of potassium ion transports enhances hypotonicity-induced cytocidal effects on GC cells, which may contribute to development of a novel lavage method for further reduction of peritoneal recurrence in GC.

## INTRODUCTION

Gastric cancer (GC) is the fourth most common cancer and the second leading cause of cancer-related death worldwide [1]. Recent advances in surgical technique and chemotherapy have improved the survival of patients with GC; however, the treatment outcome of advanced GC is still unsatisfactory because relapse frequently occurs even after potentially curative resection [2]. Peritoneal metastasis is the most common pattern of GC recurrence, and the prognosis is dismal because the effect of systemic chemotherapy on peritoneal nodules is limited due to the peritoneal-plasma barrier [2, 3].

Free cancer cells spilled into the peritoneal cavity due to tumor manipulation and lymph node dissection during surgery can be a source of peritoneal metastasis [4–9]. Thus, intraoperative peritoneal lavage plays an important role to reduce the risk of peritoneal recurrence because it can directly remove and kill such viable cancer cells. We have reported that peritoneal lavage with distilled water (DW) is a useful prophylactic treatment for peritoneal recurrence because severe hypotonic stimulation induces cell swelling followed by rupture of cancer cells [10, 11]. However, cancer cells, as well as non-cancerous cells, have the inherent potential to avoid their ruptures even under hypotonic stimulation via the mechanism of regulatory volume decrease (RVD) which involves potassium and chloride ion transports [11–17].

These findings led to the hypothesis that the inhibition of RVD by using Quinine hydrochloride (Quin), a potassium channel blocker, enhances the therapeutic effect of peritoneal lavage with hypotonic solutions. We herein examined whether blockade of potassium ion transports enhances cytocidal effects of hypotonic stimulation on GC cells via the inhibition of RVD, and hence reduces the formation of peritoneal metastasis of GC. The aim of this study was to develop a novel lavage method using a cellular physiological approach for further reduction in the incidence of peritoneal recurrence of GC after surgery.

## RESULTS

### Cell volume changes of GC cells during hypotonic stimulation

**Figure 1:**
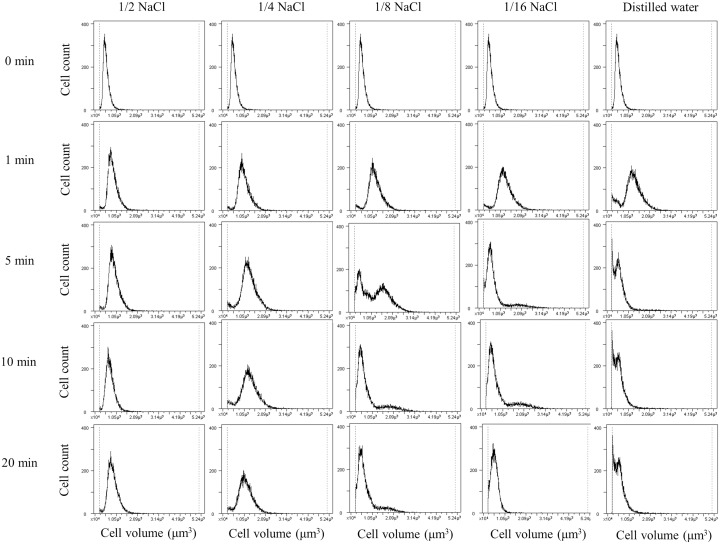
Cell volume changes of HGC-27 cells during hypotonic stimulation The cell volume and counts of HGC-27 cells were simultaneously measured at 1, 5, 10 and 20 min after hypotonic stimulation with various osmolalities. The isotonic NaCl solution was diluted 2-, 4-, 8-, or 16-fold with distilled water, and shown as 1/2, 1/4, 1/8 or 1/16 NaCl solution, respectively. The cell volume in the isotonic NaCl solution was used as a sample without hypotonic stimulation (0 min).

Serial changes in cell volume of HGC-27 cells during hypotonic stimulation of various osmolarities were shown in Figure 1. When the cells were exposed to mild hypotonic stimulation with 1/2 NaCl (approximately 150 mosmol/kgH_2_O) or 1/4 NaCl solutions (approximately 75 mosmol/kgH_2_O), the cell volume initially increased and then gradually decreased towards the original level via the mechanism known as RVD. Meanwhile, when the cells were exposed to severe hypotonic solution such as DW (0 mosmol/kgH_2_O), the cell volume showed a marked decrease following initial increase, reflecting that most cells were broken into small fragments. Similar findings were observed in Kato III and MKN45 cells, as already shown in our previous report [12].

### Cytocidal effects of DW on GC cells

**Figure 2:**
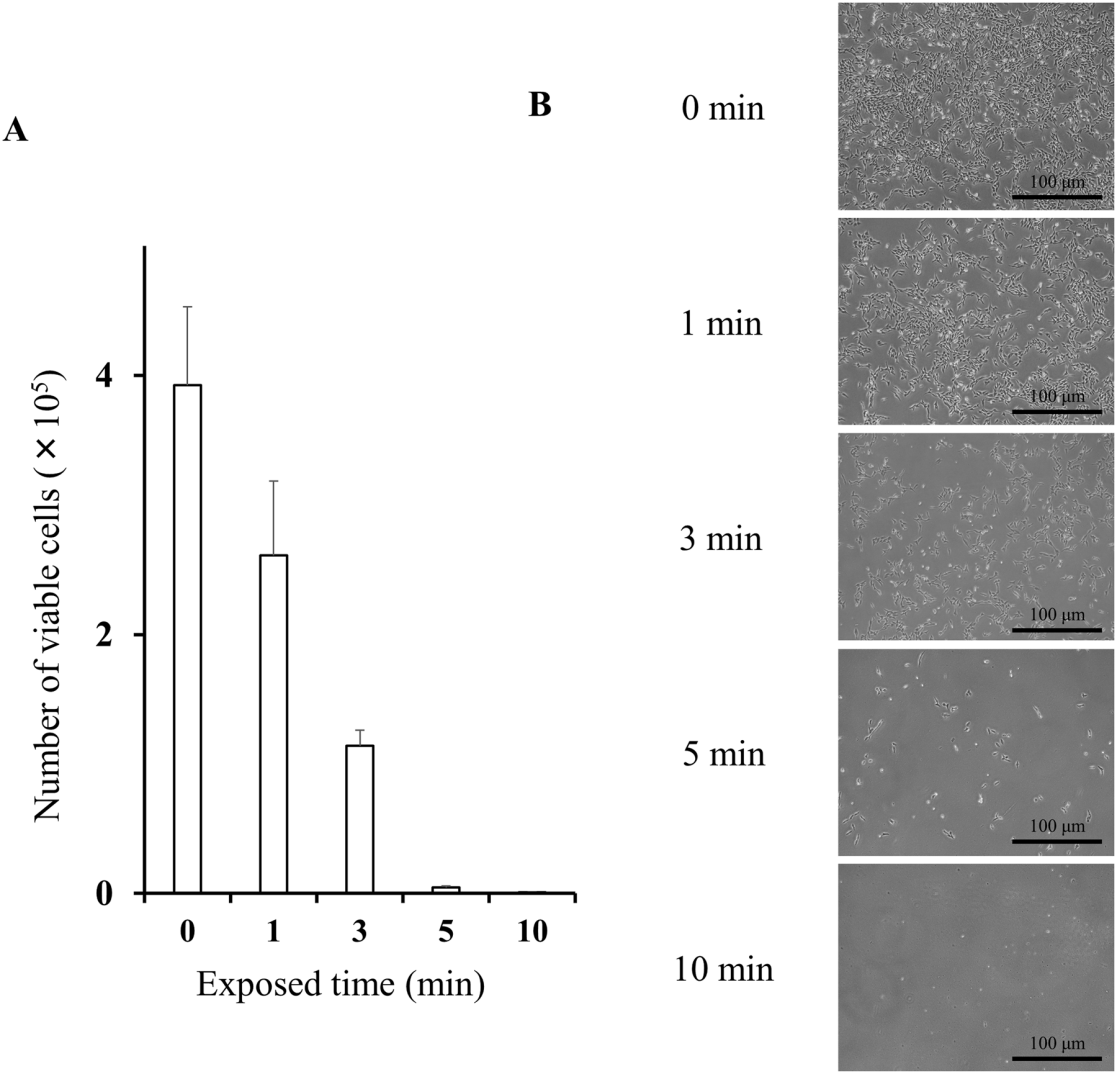
Cytocidal effects of distilled water on HGC-27 cells **(A)** The number of viable HGC-27 cells was counted 48 h after 0, 1, 3, 5, and 10 min exposure to distilled water. Data were represented as mean ± SEM (n=3). The cells suspended in the isotonic NaCl solution were used as a sample without hypotonic stimulation (0 min). **(B)** Representative pictures of cultured HGC-27 cells 48 h after 0, 1, 3, 5, and 10 min exposure to distilled water.

The number of viable HGC-27 cells counted 48 h after exposure to DW was shown in Figure 2. The number of viable HGC-27 cells decreased dependent on the exposed time to severe hypotonic stimulation with DW. Similar results were observed in Kato III and MKN45 cells, as already shown in our previous report [12].

### Blockade of potassium ion transports inhibits RVD in GC cells

**Figure 3:**
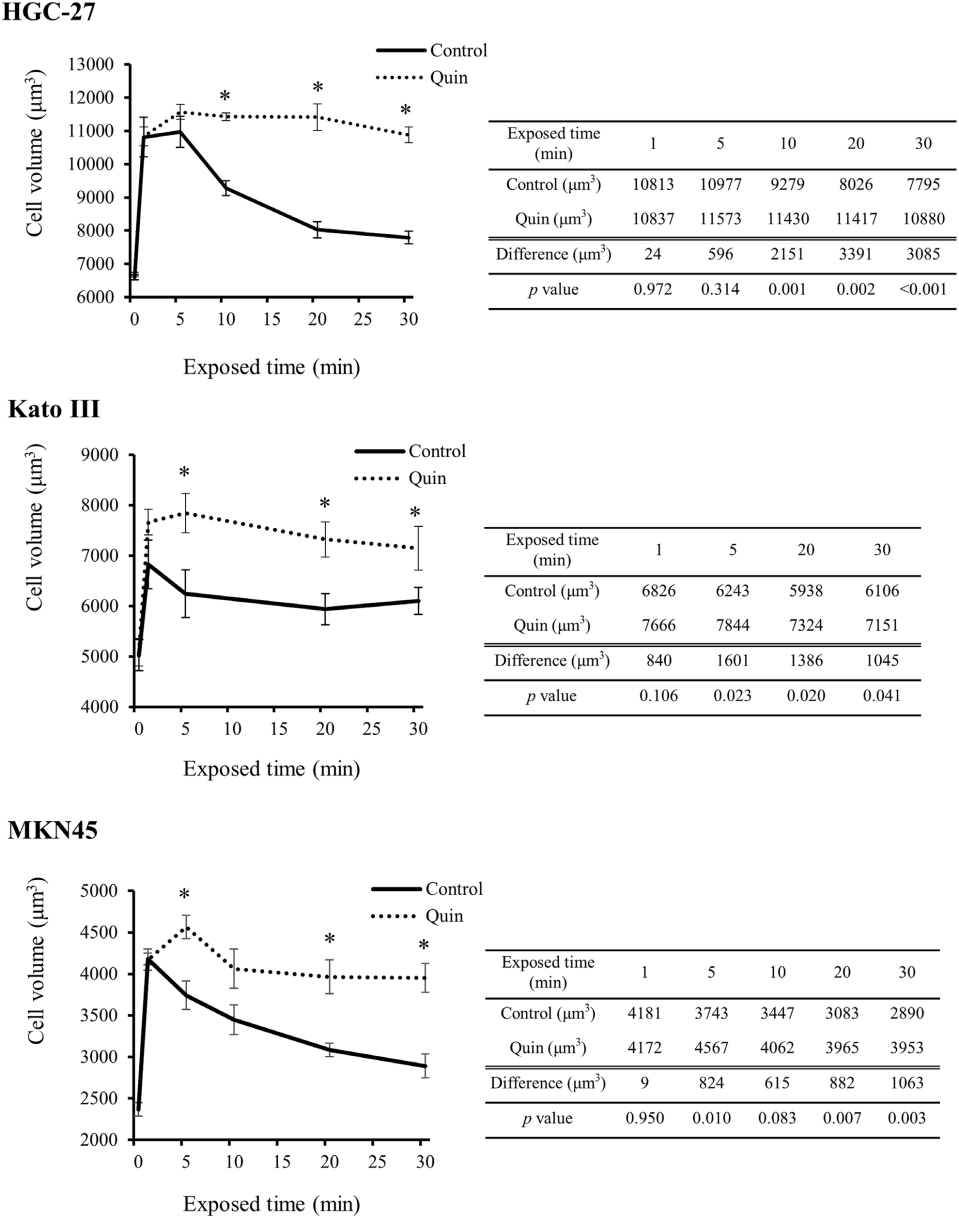
Effects of blockade of potassium ion transports on hypotonicity-induced cell volume changes in GC cells Serial changes in mean cell volume (MCV) of HGC-27, Kato III, and MKN45 cells after 1, 5, 10, 20, and 30 min exposure to the 1/2 NaCl solution with 1 mM quinine hydrochloride (Quin) or 1/2 NaCl solution alone (control) were shown. Data were represented as mean ± SEM (n=3). The cell volume in the isotonic NaCl solution with or without Quin was used as a sample without hypotonic stimulation (0 min). ^*^P < 0.05.

Figure 3 shows serial changes in mean cell volume (MCV) of GC cells during exposure to 1/2 NaCl solutions (approximately 150 mosmol/kgH_2_O) with or without 1mM Quin. When GC cells were exposed to mild hypotonic solution without Quin, the cell volume initially increased and then gradually decreased towards the original level. Meanwhile, when GC cells were treated with mild hypotonic solution containing Quin, the cells were forced to keep large in cell volume over a long time. The difference in MCV at each time point between the two groups tended to become large dependent on exposed time especially in HGC-27 and MKN45 cells. These findings clearly showed that blockade of potassium ion transports inhibited RVD in GC cells.

### Blockade of potassium ion transports enhances cytocidal effects of hypotonic stimulation on GC cells

**Figure 4:**
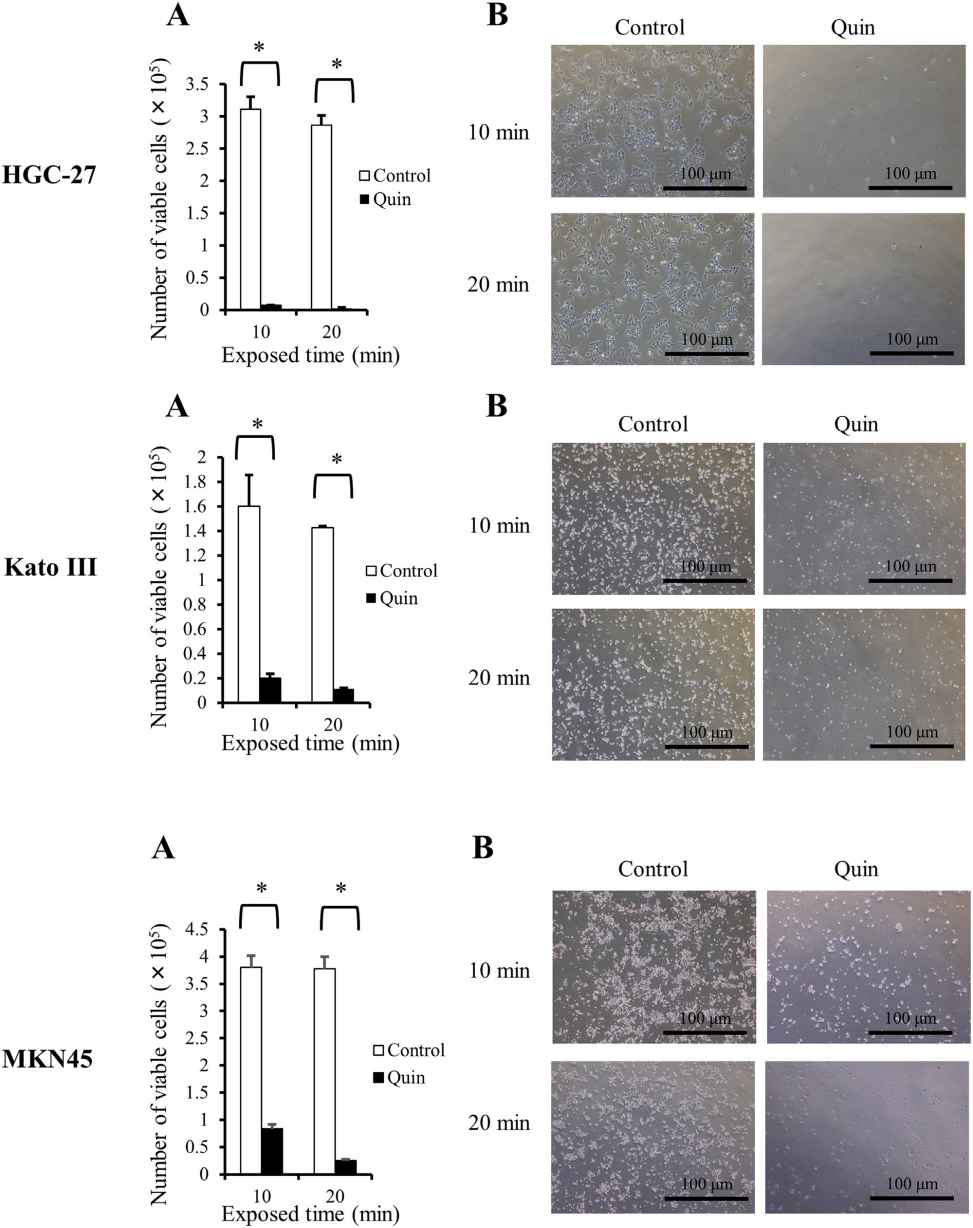
Influences of blockade of potassium ion transports on cytocidal effects of hypotonic stimulation in GC cells **(A)** The number of viable HGC-27, Kato III, and MKN45 cells was counted 48 h after 10 or 20 min exposure to 1/4 NaCl solution with 1 mM quinine hydrochloride (Quin) or 1/4 NaCl solution alone (control). Data were represented as mean ± SEM (n=3). ^*^P < 0.05. **(B)** Representative pictures of cultured GC cells 48 h after 10 or 20 min exposure to 1/4 NaCl solution with Quin (Quin) or 1/4 NaCl solution alone (control).

**Figure 5:**
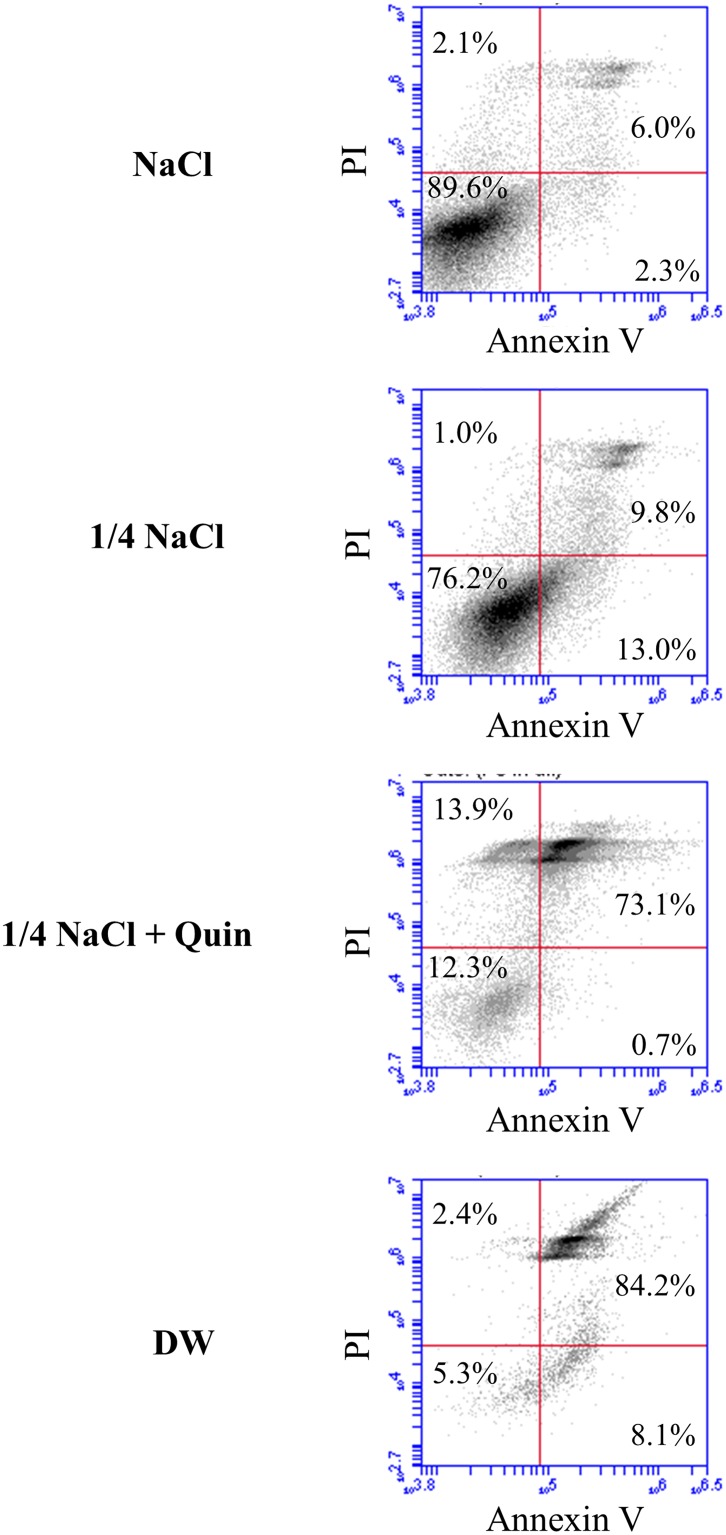
Apoptosis assay in HGC-27 cells treated with hypotonic stimulation Representative data of apoptosis assay in HGC-27 cells treated with isotonic NaCl solution, 1/4 NaCl solution alone, 1/4 NaCl solution containing 1 mM Quin, or DW.

The number of viable GC cells counted 48 h after 10 or 20 min exposure to 1/4 NaCl solution (approximately 75 mosmol/kgH_2_O) with or without 1mM Quin was shown in Figure 4. In all of three GC cell lines, mild hypotonic stimulation with Quin significantly reduced the number of viable cells compared to mild hypotonic stimulation alone; thus, blockade of potassium ion transports effectively enhanced cytocidal effects of hypotonic stimulation on GC cells. Apoptosis assays in HGC-27 cells showed that blockade of potassium ion transports clearly increased mild hypotonicity-induced dead cells due to cell ruptures, but did not induce early apoptosis (Figure 5).

### Blockade of potassium ion transports enhances the therapeutic effect of peritoneal lavage with hypotonic solution: *in vivo* study

**Figure 6:**
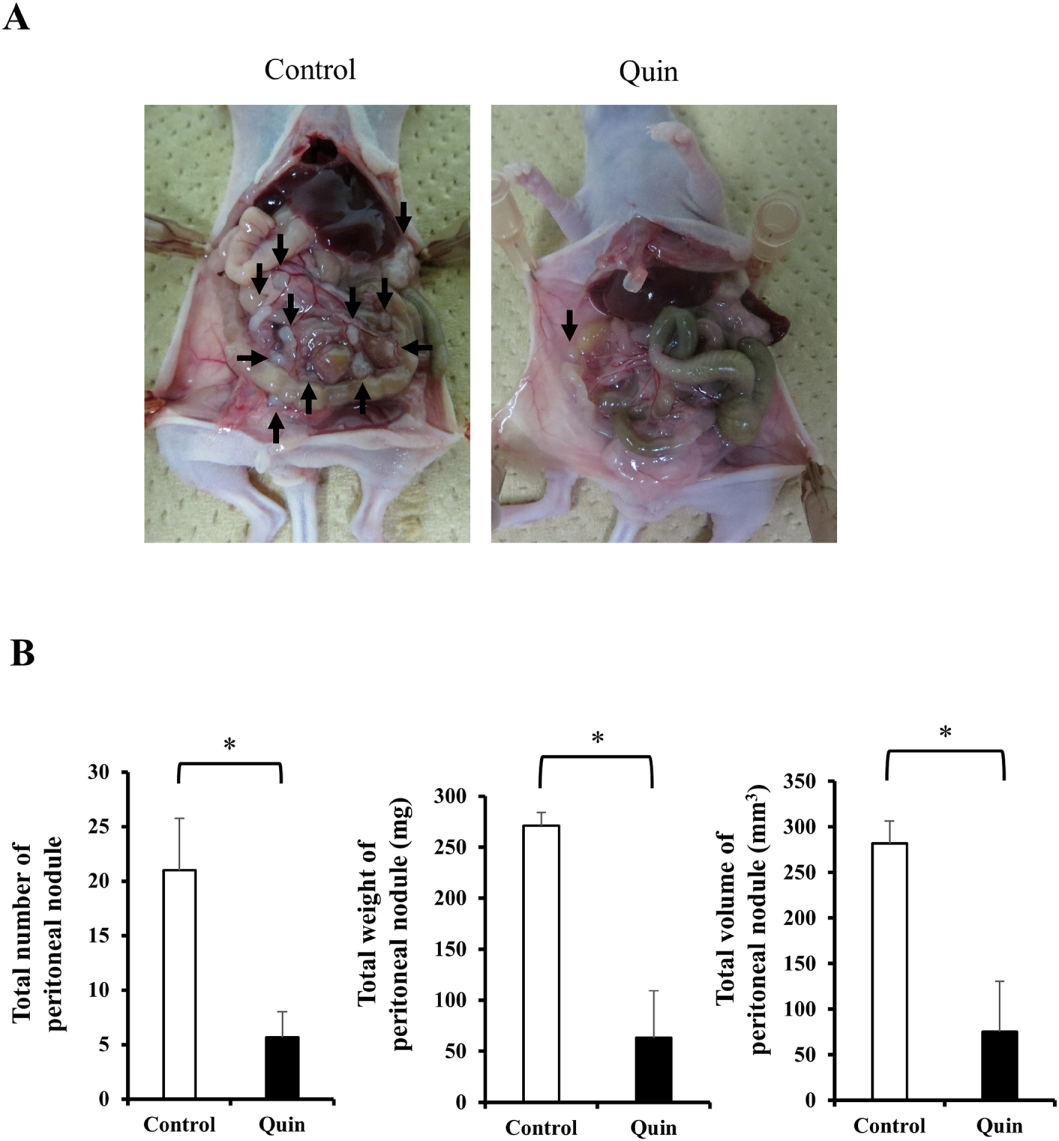
Effects of blockade of potassium ion transports during hypotonic stimulation on the formation of peritoneal metastases of MKN45 cells in nude mice **(A)** Representative macroscopic findings of established peritoneal nodules 2 weeks after peritoneal injection of MKN45 cells stimulated with 1/4 NaCl solution containing 1 mM quinine hydrochloride (Quin) or 1/4 NaCl solution alone (control) were shown. The *arrows* pointed peritoneal nodules. **(B)** The total number, total weight, and total volume of established peritoneal nodules were compared between the Quin and the control groups. Data were represented as mean ± SEM (n=3). ^*^P < 0.05.

Representative macroscopic findings of established peritoneal nodules in nude mice were shown in Figure 6A. Only a few peritoneal nodules were observed when MKN45 cells had been treated with 1/4 NaCl solution (approximately 75 mosmol/kgH_2_O) containing Quin (Quin group), while many peritoneal nodules were established when MKN45 cells had been stimulated with 1/4 NaCl solution alone (control group). The comparative data of the total number, total weight, and total volume of established peritoneal nodules in nude mice were shown in Figure 6B. The total number of established peritoneal nodules was significantly less in the Quin group (5.7 ± 2.3) than in the control group (21.0 ± 4.7) (*p* = 0.044). Also, the total weight and total volume of established peritoneal nodules were significantly lower in the Quin group (63.0 ± 46.4 mg, 75.3 ± 55.5 mm^3^) than in the control group (271.0 ± 13.1 mg, 281.9 ± 24.8 mm^3^) (*p* = 0.013, and 0.027, respectively).

## DISCUSSION

Formation of peritoneal metastasis of GC consists of a multistep process, but the details of underlying molecular mechanisms remain largely unclear [18–21]. The detachment of cancer cells from the serosa of primary tumor followed by their attachment to peritoneal mesothelial cells is thought to be crucially important processes for metastasis formation. Meanwhile, recent studies have shown that cancer cell spillage occurs during surgery due to tumor manipulation or lymph node dissection, and such viable cancer cells can also be sources of peritoneal metastasis [4–9]. Therefore, effective intraoperative peritoneal lavage is important to prevent peritoneal recurrence of GC because it can directly remove and/or kill these viable cancer cells before their implantation on the peritoneum.

The lavage method based on the ‘limiting dilution theory’ may be one of the useful intraoperative techniques to prevent the implantation of free GC cells on the peritoneum. Kuramoto et al. previously reported a considerable reduction in peritoneal recurrence with extensive intraperitoneal lavage (EIPL) in advanced GC patients with intraperitoneal free cancer cells without overt peritoneal metastasis (CY+/P-) [22]. In this technique, the peritoneal cavity is extensively washed and completely aspirated using 1L of physiological saline 10 times with the aim to dilute the number of free cancer cells to potentially zero. However, even with such a useful lavage technique, about half of patients with CY+/P- still experienced peritoneal recurrence of GC: thus, more effective lavage method had better be employed which can eradicate free cancer cells remaining persistently in the peritoneal cavity even after extensive lavage.

Peritoneal lavage with DW may be another useful intraoperative therapy due to its cytocidal potential, and has been used for surgeries of various cancers [23–25]. We have so far succeeded in observing changes in cellular morphology and volume of GC cells subjected to DW using several unique methods and apparatus [12]. Video recordings using a digital camera (30 frames/sec, 512 × 512 pixels, 10 bits per pixel), and measurements of cell volume changes using a high-resolution flow cytometer clarified that hypotonic stimulation with DW induced swelling followed by rupture of GC cells within 5 min [12]. When GC cells were incubated with DW, the number of viable cells actually decreased in an exposure time dependent manner. Furthermore, in the *in vivo* model, 20 min incubation of MKN45 cells with DW, instead of saline, markedly inhibited the formation of peritoneal nodules in nude mice, whereas the microscopic findings did not show any histological damages in the peritoneal mesothelial cells [10].

On the other hand, our previous study also demonstrated that the actual osmolality of lavage fluid collected after peritoneal lavage with DW during GC surgery was approximately 50 mosmol/kgH_2_O probably due to the contamination of secretions and various cell lysates in the peritoneal cavity [12]. Therefore, such an elevation in osmolality of lavage fluid might attenuate the cytocidal effects of hypotonic stimulation with DW, and, thus, some GC cells can avoid their ruptures via the mechanism known as RVD. That is, GC cells, as well as non-cancerous cells, could return to their original volume following their initial swelling by water transports via the activation of potassium and chloride channels [11–17]. During RVD, the intracellular concentrations of potassium and chloride ion decrease, and intracellular water moves to the extracellular side [16].

To overcome an assumed attenuation in cytocidal effects of peritoneal lavage with DW in actual surgeries, we tried to inhibit the occurrence of RVD using Quin, a potassium channel blocker which is utilized mainly for the chemoprophylaxis of malaria. Considering the actual osmolality of lavage fluid after peritoneal lavage with DW, 1/4 NaCl solution (approximately 75 mosmol/kgH_2_O), instead of DW, was used to examine the effects of blockade of potassium ion transports on cytocidal effects on GC cells. To the best of our knowledge, the present study was the first to examine the effects of blockade of potassium ion transports during mild hypotonic stimulation on cell volume changes and cytocidal effects on GC cells. As a result, blockade of potassium ion transports inhibited the occurrence of RVD, and hence markedly enhanced cytocidal effects of hypotonic stimulation on GC cells. Furthermore, the formation of peritoneal metastases in nude mice was effectively prevented by the incubation of free GC cells with mild hypotonic solutions containing a potassium channel blocker. These findings clearly indicated that blockade of potassium ion transports may contribute to keeping the strong cytocidal effects of intraoperative peritoneal lavage with DW on free GC cells even if the osmolality of lavage fluid slightly increases due to the contamination of secretions and various cell lysates in the peritoneal cavity.

There were some limitations in this study. Our experimental design of *in vivo* model was not sufficient enough to show the usefulness of intraoperative lavage with hypotonic solution containing a potassium channel blocker because most GC cells incubated with Quin should have been dead before their peritoneal injection. Another experimental design might solve the weakness of our work where nude mice are treated with intraperitoneal injection of hypotonic solutions with Quin after injection of GC cells; however, such a method carries a difficulty in collecting the injected hypotonic solutions, that is also different from the situation of actual intraoperative peritoneal lavage. Although the therapeutic effects for already established peritoneal nodules could not be clarified, the present study clearly showed that cytocidal effects of mild hypotonic shock with Quin were sufficient for isolated free GC cells.

Another concern is damages in normal mesothelial cells or peritoneal tissues by hypotonic stress, even though the present study aimed to target free GC cells alone. Regarding the difference in cytocidal effects between cancer and non-cancerous cells, our previous study have already shown that pancreatic cancer cells were more sensitive to hypotonic shock than human lung fibroblast WI38 cells [15]. Meanwhile, our previous *in vivo* experiments have also shown that peritoneal injection of DW did not severely damage the peritoneum and abdominal organs of mice, suggesting its safeness in an *in vivo* use [10]. Recently, many researchers including ours have reported the expression and role of ion and water channels/transporters for cancer progressions, which also involve in regulating cell volume [12, 26–29]. Actually, there are clear differences in hypotonicity-induced cell volume changes and cytocidal effects among cancer cell lines or between cancer cells and non-cancerous cells [11–15]; thus, further investigations of specific ion and water channels/transporters would contribute to development of novel therapeutic strategy for the prevention of peritoneal metastasis.

In conclusion, blockade of potassium ion transports during mild hypotonic stimulation enhances cytocidal effects on GC cells via the inhibition of RVD, and hence reduces the formation of peritoneal metastasis of GC. Therefore, our novel lavage method using a cellular physiological approach may contribute to further reduction in peritoneal recurrence of GC after curative surgery.

## MATERIALS AND METHODS

### Cell culture and materials

The human GC cell lines HGC-27, Kato III, and MKN45 were purchased from RIKEN BioResource Center, Tokyo, Japan. These cell lines were maintained in RPMI medium (Nacalai Tesque, Kyoto, Japan) supplemented with 10% fetal bovine serum, 100 U/ml of penicillin and 100 μg/ml of streptomycin. The flasks were kept in a humidified incubator at 37°C with 5.0% CO^2^ in air. Quin, a potassium channel blocker, was purchased from Nacalai Tesque.

### Preparation of isotonic and hypotonic NaCl solutions

The 140 mM NaCl isotonic solution contained 140 mM NaCl, 5.0 mM KCl, 1.0 mM CaCl_2_, 1.0 mM MgCl_2_, 5.0 mM glucose, and 10 mM HEPES. The pH of each solution was adjusted to 7.4 with NaOH. The osmolality of the solution was measured using a freezing point osmometer (model 110, Fiske Associates, Norwood, MA), and determined as 300 mosmol/kgH_2_O for the isotonic NaCl solution, and 0 mosmol/kgH_2_O for DW. Autoclaved Milli-Q water was used for our DW working solution [10–15]. Graded hypotonic NaCl solutions were made by diluting the stock isotonic NaCl solution 2-fold, 4-fold, 8-fold and 16-fold with DW. For example, 1/2 NaCl solution meant NaCl solution diluted 2-fold with DW, and, thus, the osmolarity was about half of isotonic NaCl solution.

### Measurement of cell volume changes of GC cells during hypotonic stimulation

Cell volume was measured by a high-resolution flow cytometer, Cell Lab Quanta (Beckman Coulter, Fullerton, CA, USA), according to a previously reported procedure [11–15]. Briefly, a total of 1.0 × 10^6^ pelleted GC cells were suspended in 1 ml of hypotonic NaCl solutions or DW. The cell suspensions were subsequently displaced into a Vi-CELL™ Sample Cup (Beckman Coulter), and cell volumes were immediately measured at a fixed time after hypotonic stimulation. The cell volume in the isotonic NaCl solution was used as a sample without hypotonic stimulation (0 min).

In the potassium channel regulation experiments, GC cells were pre-treated with the isotonic NaCl solution containing 1 mM Quin for 15 min at room temperature (20-24°C). Then, cell volumes were measured at a fixed time after hypotonic stimulation with 1/2 NaCl solution (approximately 150 mosmol/kgH_2_O) containing 1 mM Quin, which was the best to examine the true effects of blockade of potassium ion transports on RVD.

### Evaluation of cytocidal effects of hypotonic stimulation on GC cells

The detailed method of re-incubation experiments to evaluate cytocidal effects of hypotonic stimulation was described in our previous studies [11–15]. Briefly, a total of 2.0 × 10^5^ GC cells were suspended in hypotonic NaCl solutions or DW for a fixed time. Then, the cell suspension was centrifuged, and the pelleted cells were re-suspended in culture medium and seeded into 6 well plates. At a set time of 48 h after plating, the number of viable cells was counted using Trypan blue and the Countess^®^ Automated Cell Counter (Invitrogen, Tokyo, Japan). The cells suspended in the isotonic NaCl solution were used as a sample without hypotonic stimulation (0 min).

In the potassium channel regulation experiments, a total of 2.0 × 10^5^ GC cells were pre-treated with the isotonic NaCl solution containing 1 mM Quin for 15 min at room temperature. The cells were then exposed to 1/4 NaCl solution (approximately 75 mosmol/kgH_2_O) containing 1 mM Quin for 10 or 20 min at room temperature. Thereafter, the cell suspension was centrifuged, and the pelleted cells were re-suspended in culture medium and seeded into 6 well plates. At a set time of 48 h after plating, the number of viable cells was counted.

### Analysis of apoptosis cells

HGC27 cells were harvested after 10 min stimulation with isotonic NaCl solution (300 mosmol/kgH_2_O), 1/4 NaCl solution (approximately 75 mosmol/kgH_2_O), 1/4 NaCl solution containing 1 mM Quin, or DW (0 mosmol/kgH_2_O), and then double-stained with fluorescein isothiocyanate-conjugated Annexin V and phosphatidylinositol (PI) using the Annexin V kit (Beckman Coulter, Brea, CA, USA) according to the manufacturer's protocols. Becton-Dickinson Accuri C6 FACS was used to analyze the proportion of apoptotic cells.

### *In vivo* experiments

Four-week-old female BALB/c nude mice purchased from SHIMIZU Laboratory Supplies Co., Ltd, (Kyoto, Japan) were maintained under pathogen-free barrier conditions. Mice were provided with sterile food and water, and housed in cages. Ambient light was controlled to provide regular 12 h light-dark cycles. All animal protocols were approved by the institutional guidelines of the Kyoto Prefectural University of Medicine, Kyoto, Japan.

MKN45 cells were used to examine whether blockade of potassium ion transports during hypotonic stimulation reduced the formation of peritoneal metastasis of GC in nude mice. A total of 1.0 × 10^6^ MKN45 cells were incubated in 5 ml of 1/4 NaCl solution (75 mosmol/kgH_2_O) with or without 1 mM Quin for 20 min. After stimulation, the cell suspension was centrifuged and re-suspended in 0.3 ml of PBS, and then intraperitoneally injected into nude mice (n=3, each group). All mice were sacrificed 2 weeks after the intraperitoneal injection of MKN45 cells, and the formation of peritoneal nodules was macroscopically evaluated. All tumors larger than 0.5 mm in diameter were resected and counted, and their diameter and weights were measured. Tumor volumes were calculated as follows [10, 11, 30]: tumor volume = length × width^2^ × 0.5.

### Statistical analysis

Statistical analyses were carried out using JMP 10 (SAS Institute, Cary, NC, USA). The cell volume and counts in *in vitro* studies, and the number, weight, and volume of peritoneal nodules in *in vivo* studies were expressed as mean ± standard error of the mean (SEM). Differences between the two groups were analyzed using the Student's *t*-test, and were considered to be significant when the *p* value was less than 0.05.
